# Time-dependent diffusion MRI for noninvasive molecular subtype differentiation and biological correlation in breast cancer: emphasizing the emerging three-tier HER2 classification

**DOI:** 10.3389/fonc.2025.1739008

**Published:** 2026-01-14

**Authors:** Mingzhe Xu, Kuiyuan Liu, Shouning Zhang, Haotian Li, Renzhi Zhang, Chunmiao Xu, Junhui Yuan, Yue Wu, Dan Wu, Xuejun Chen, Jinrong Qu

**Affiliations:** 1 The Department of Radiology, The Affiliated Cancer Hospital of Zhengzhou University & Henan Cancer Hospital, Zhengzhou, China; 2 The Department of Biomedical Engineering, College of Biomedical Engineering & Instrument Science, Zhejiang University, Hangzhou, Zhejiang, China; 3 The Department of Radiology, National Cancer Center/National Clinical Research Center for Cancer/Cancer Hospital, Chinese Academy of Medical Sciences and Peking Union Medical College, Beijing, China

**Keywords:** breast cancer, HER2 expression, molecular subtypes, subtype differentiation, time-dependent diffusion-weighted imaging

## Abstract

**Background:**

Breast cancer is a heterogeneous disease, and accurate subtype characterization is essential for guiding personalized treatment. In particular, HER2-low tumors have recently emerged as a distinct clinical entity with potential responsiveness to novel HER2-targeted therapies. However, reliable noninvasive imaging methods to identify these subgroups remain lacking.

**Purpose:**

To evaluate the potential of time-dependent diffusion MRI (T_d_-dMRI) in differentiating breast cancer molecular subtypes and to investigate its correlation with immunohistochemical biomarkers, particularly the newly established three-tier HER2 classification.

**Materials and methods:**

In this retrospective study, female patients with untreated invasive ductal carcinoma underwent 3T breast MRI including T_d_-dMRI between June 2023 and October 2024. A custom protocol combining oscillating gradient spin-echo (OGSE) and pulsed gradient spin-echo (PGSE) sequences enabled diffusion sampling at multiple diffusion times and frequencies. Microstructural parameters—cellularity, extracellular and intracellular diffusivity (D_ex_, D_in_), cell diameter, intracellular volume fraction (f_in_), and intracellular water residence time (τ_in_)—were estimated using a Bayesian model based on a joint multicompartmental framework. Molecular subtypes (Luminal A/B, HER2-enriched, triple-negative [TN]) and HER2 expression levels (HER2-zero, HER2-low, HER2-positive) were determined via IHC and fluorescence *in situ* hybridization (FISH). Quantitative T_d_-dMRI metrics were compared across subtypes and correlated with ER, PR, HER2, and Ki-67 status using ANOVA, Kruskal–Wallis, and ROC curve analysis.

**Results:**

This study included 71 female participants (mean age, 51.3 ± 10.2 years). Multiple T_d_-dMRI parameters varied significantly across molecular and HER2 subtypes. ADC_50Hz_ was significantly higher in Luminal A compared to Luminal B (P = 0.003). HER2-enriched tumors showed higher ADC values and cell diameters but lower cellularity compared to Luminal B (P< 0.05). ER− and PR− tumors had higher ADCs, cell diameters, and D_in_, with lower cellularity than positive counterparts. D_in_ effectively distinguished TN from non-TN cancers (AUC = 0.710). For HER2 stratification, ADC_30ms_ distinguished HER2-zero from HER2-low tumors with high accuracy (AUC = 0.898), and cell diameter and cellularity were most effective for differentiating HER2-low from HER2-positive tumors (AUC = 0.770). No significant T_d_-dMRI differences were observed for Ki-67.

**Conclusion:**

ADC_30ms_ most effectively distinguished HER2-zero from HER2-low tumors, while microstructural parameters such as cellularity and cell diameter moderately differentiated HER2-low from HER2-positive cancers. These results support the potential of T_d_-dMRI as a complementary imaging biomarker for subtype characterization, although findings were limited by small subgroup sizes and the single-center design.

## Introduction

Breast cancer is a biologically heterogeneous disease comprising distinct molecular subtypes—luminal A, luminal B, human epidermal growth factor receptor 2 (HER2) -enriched, and triple-negative (TN)—defined by immunohistochemical (IHC) markers including estrogen receptor (ER), progesterone receptor (PR), HER2, and Ki-67. These markers are essential for diagnosis, treatment selection, and prognostic assessment. Therefore, noninvasive methods that can accurately characterize these subtypes and markers are crucial for optimizing personalized therapy.

Breast magnetic resonance imaging (MRI) is recommended by the American College of Radiology (ACR) as an important modality for breast cancer screening, diagnosis, and treatment monitoring (1). Diffusion-weighted imaging (DWI) is widely used for its sensitivity to tissue microstructure by detecting water molecule motion. Advanced diffusion models, such as diffusion tensor imaging (DTI), diffusion kurtosis imaging (DKI), and intravoxel incoherent motion (IVIM), provide additional microstructural and perfusion insights by varying gradient directions or b-values. However, these models assume a fixed diffusion time, and their quantitative parameters often yield overlapping values (e.g., ADC value) among molecular subtypes (2), limiting their ability to reflect specific microstructural features of tumor pathology.

Time-dependent diffusion MRI (T_d_-DWI) overcomes this limitation by varying diffusion times or oscillation frequencies to probe water diffusion across temporal scales. Short diffusion times reflect restricted movement within intracellular or subcellular environments, while longer times are more sensitive to extracellular spaces and overall tissue architecture (3). Consequently, T_d_-DWI can reveal structural features such as cell size, membrane permeability and extracellular matrix complexity (4), and has demonstrated high sensitivity to tumor microscopic pathological features at cellular and even subcellular scales in previous *in vitro* cellular and animal studies (5–7). Although applied in head and neck tumors (8), breast cancer (9) and prostate cancer (4), T_d_-DWI’s clinical application in human tumors remains preliminary. Su et al. recently demonstrated its feasibility in differentiating benign from malignant breast lesions (10); however, its value in distinguishing molecular subtypes and IHC profiles is still underexplored.

A major motivation for this study arise from the recent refinement of the HER2 classification, with the emergence of HER2-low—defined as IHC 1+ or IHC 2+ without fluorescence *in situ* hybridization (FISH) amplification—as a distinct third category (11). HER2-low tumors, accounting for approximately 55% of breast cancers, are emerging as a therapeutically relevant subgroup, responding to novel HER2-targeted agents such as antibody-drug conjugates (11–13). These tumors also exhibit distinct biological and clinicopathologic characteristics compared to HER2-zero and HER2-positive cancers (13, 14). Accurate identification of HER2-low lesions is therefore essential but remains challenging. Conventional pathology-based classification relies on limited biopsy samples and is susceptible to variability in staining quality, reader interpretation, and tissue sampling—issues that may lead to misclassification, particularly among borderline HER2-low cases. These constraints highlight an unmet clinical for complementary, noninvasive imaging biomarkers capable of capturing whole-tumor biology and improving HER2 stratification (15). Besides, as global incidence of breast cancer continues to rise and pathology resources vary across medical settings, accessible imaging methods that enhance subtype detection could help support more equitable treatment decisions (16). At the same time, emerging HER2-targeted therapies place increasing demands on accurate subtype characterization, underscoring the technological importance of developing diffusion-based imaging tools that reflect tumor microstructure *in vivo*.

Therefore, this study aimed to investigate the potential of T_d_-DWI–derived microstructural parameters in distinguishing molecular subtypes of breast cancer and in assessing their correlation with key immunohistochemical markers, with particular emphasis on the updated three-tier HER2 classification.

## Materials and methods

### Patients

**Figure 1:**
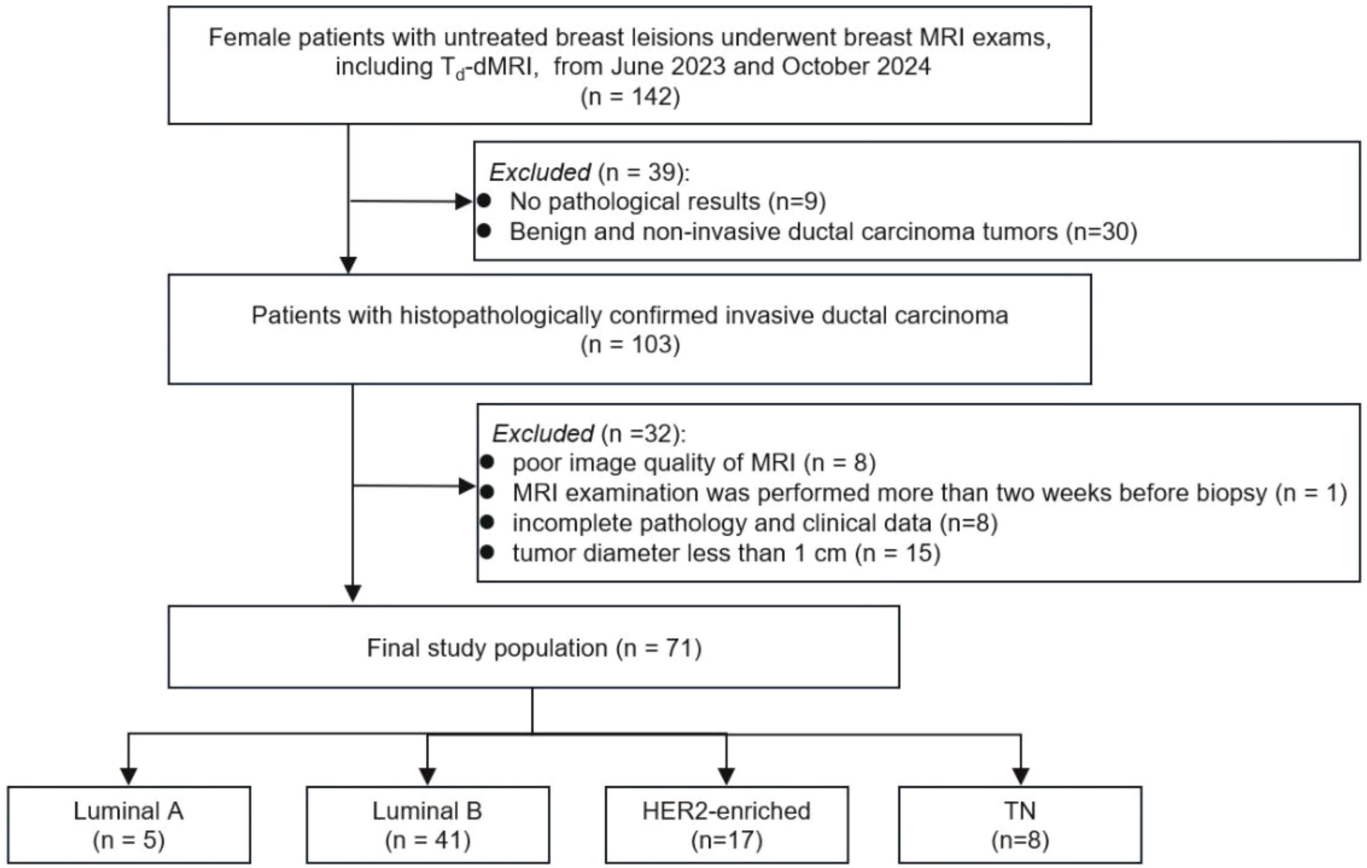
Flowchart of the patient selection process for the study cohort. T_d_-dMRI, time-dependent diffusion MRI; TN, triple-negative.

This study was approved by the Institutional Review Committee of the Affiliated Cancer Hospital of Zhengzhou University (No.2025-281), with a waiver for informed consent. Consecutive 103 female patients with untreated breast invasive ductal carcinoma patients (mean age 51.3 ± 10.2 years) who underwent pretreatment breast MRI including T_d_-dMRI between June 2023 and October 2024 were enrolled in this study. Furthermore, 32 patients were excluded because of following reasons: 1) poor image quality of MRI (n = 8), 2) MRI examination was performed more than two weeks before biopsy (n = 1), 3) incomplete pathology and clinical data (n=8), 4) tumor diameter less than 1 cm (n = 15). Ultimately, 71 patients were classified into four molecular subtypes: luminal A (n = 5), luminal B (n = 41), HER2-enriched (n = 17), and TN (n = 8). The study flowchart was shown in **Figure 1**.

In this study, 38 patients underwent surgical resection after neoadjuvant chemotherapy (NAC). Based on the surgical pathology findings according to the St. Gallen International Breast Cancer Consensus (17), pathological complete response (pCR) was defined as the absence of residual invasive carcinoma in the primary breast lesion (ypT0) with no regional lymph node metastasis (ypN0) in the surgical specimen.

Treatment protocols were individualized based on immunohistochemical profiles (e.g., HER2 status, ER/PR expression) and genetic characteristics of biopsy samples. Regimens included: TCbHP (docetaxel + carboplatin + trastuzumab + pertuzumab), TCb (docetaxel + carboplatin), TCH (docetaxel + carboplatin + trastuzumab), PH (paclitaxel + trastuzumab) combined with pyrotinib, and TE (docetaxel + epirubicin). Each treatment cycle lasted 21 days, with a total of 6 to 8 cycles administered.

### MRI acquisition protocol

All imaging was conducted on a 3T MRI scanner (Prisma, Siemens Healthineers, Germany). The standard MRI protocol included axial T1WI (repetition time [TR] = 5.36 ms, echo time [TE] = 2.46 ms, matrix = 374 × 416, field of view [FOV] = 416 × 416 mm², slice thickness = 1.5 mm, slice gap = 0 mm, scanning time of approximately 58 s), fat-suppressed T2WI (TR/TE = 8870/90 ms, matrix = 358 × 448, FOV = 340 × 340 mm², slice thickness/gap = 4/4.4 mm, acquisition time of approximately 2 min 19 s), axial dynamic contrast-enhanced T1-weighted images (TR/TE = 4.01/1.34 ms, matrix = 214 × 320, FOV = 320 × 320 mm², slice thickness/gap = 1.5/0 mm, flip angle = 9°, scanning time of approximately 5 min 32 s including 40 phases) and sagittal delayed phase images. To assess the time-dependency of water diffusion in tissue microstructure, a custom T_d_-dMRI protocol was implemented. This protocol combined an in-house oscillating gradient spin-echo (OGSE) sequence using a trapezoid cosine gradient waveform with a conventional pulsed gradient spin-echo (PGSE) sequence (18). Together, these sequences enabled diffusion measurements across a range of effective diffusion times. OGSE acquisitions were performed at two frequencies: 50 Hz (effective diffusion time = 5 ms; 2 cycles; b = 250 and 500 s/mm²) and 25 Hz (effective diffusion time = 10 ms; 1 cycle; b = 250, 500, 1000, and 1500 s/mm²). PGSE acquisitions were performed at: diffusion separation time (Δ) = 30 ms (effective diffusion time = 26.67 ms; b = 250, 500, 1000, and 1500 s/mm²),and Δ = 50 ms (effective diffusion time = 46.67 ms; b = 250, 500, 1000, and 1500 s/mm²). The remaining scan parameters were consistent across all diffusion sequences: TR = 4100 ms, TE = 106 ms, FOV = 300 × 300 mm², matrix size = 80 × 80, and slice thickness = 5 mm. For each b-value, diffusion gradients were applied along six directions, with a single non-diffusion-weighted (b = 0) image acquired per slice. The total scan duration for the complete time-dependent diffusion MRI protocol was approximately 5 min 11 s.Dynamic contrast-enhanced T1-weighted images were acquired using a fat-saturated gradient-echo sequence following intravenous administration of gadoteric acid (Gd-DOTA) at a standardized dose of 0.1 mmol/kg, delivered via a power injector at a flow rate of 1.5 mL/s.

### Image processing and analysis

For conventional diffusion analysis, ADC maps were generated using monoexponential fitting at four diffusion conditions (ADC_50ms_, ADC_30ms_, ADC_25Hz_, and ADC_50Hz_) derived from different combinations of diffusion times and oscillation frequencies. Microstructural quantification was estimated using nonlinear least-squares (NLLS) fitting based on a multicompartmental joint model that integrated the modified Kärger model and IMPULSED method, incorporating intra-cellular, extracellular, and transcytolemmal water exchange effects, as proposed by Jiang et al. (3, 18). This model enables the estimation of detailed tissue microstructure by resolving diffusion behavior across cellular compartments and membrane interfaces. Fitting was performed in MATLAB R2018a (Mathworks, Natick, MA) using the lsqcurvefit function with a trust-region–reflective algorithm (18). Fitted parameters were: cellularity, extracellular diffusivity (D_ex_), intracellular diffusivity (D_in_), cell diameter, intracellular volume fraction (f_in_), and intracellular water residence time (τ_in_). The cellularity was defined as f_in_/cell diameter.100 for simplicity. Consistent with prior work (18), the D_in_ was fixed at 1.0 μm²/ms considering the fitting was insensitive to the choice of intracellular diffusivity (19). The optimization minimized the squared difference between the IMPULSED output and the measured signal (Equation 1), where n denotes the number of measured T_d_-dMRI signals, and $seqParams_{i}$ represents the sequence parameters in PGSE and OGSE, including gradient duration ( δ), diffusion interval ( Δ), gradient field strength ( g), rise time ( tr), and number of waveform cycles (N). S and $S_{i}$ denote the IMPULSED output and the signal measured under a specific sequence parameter set, respectively. To achieve the global optimum, the NLLS fitting was repeated 100 times using randomized initial values within physiologically plausible bounds: 0< f_in_< 1, 1< cell diameter< 40 μm, and 0.5< D_ex_< 3.5 μm²/ms (18). The final estimates were selected from the iteration yielding the minimal residual error.

(1) $$\underset{f_{in},d,D_{ex}}{\min}\sum_{i=1}^{n}(S(f_{in},d,D_{ex},seqParams_{i})-S_{i}(seqParams_{i}){)}^{2}$$

To improve the robustness and accuracy of parameter estimation under clinical acquisition constraints, we adopted a Bayesian model fitting strategy as developed by Liu et al. (18), This method utilized prior information and posterior probability distributions to constrain parameter space and reduce estimation uncertainty, especially in the presence of noise and limited diffusion sampling. According to the methodology described in Liu’s previous work (18), numerical simulations were also performed to assess the robustness of the model and to validate the experimental procedures. The detailed simulation methods, parameter settings, and analytical formulas were available in Liu’s published study (18).

Using the MR segmentation software ITK-SNAP, three-dimensional (3D) regions of interest (ROIs) were manually delineated on contrast- arterial phase contrast-enhanced T1-weighted images by two radiologists (with 10 and 15 years of experience in breast MRI, respectively) and propagated to all T_d_-dMRI maps. Only solid tumor regions were included, carefully avoiding necrotic, hemorrhagic, and cystic components. Two radiologists also independently evaluated the breast MRI examinations using the Breast Imaging Reporting and Data System criteria, with strict blinding to lesion locations, histopathological diagnoses, and clinical data. Discrepancies were resolved by consensus.

### Biological status assessment

Histopathological evaluation of lesion specimens was conducted by 2 pathologists with 6 and 15 years of specialized breast pathology experience, respectively. Through consensus review of hematoxylin-eosin staining and immunohistochemical profiles, any disagreements were resolved through discussion in accordance with WHO guidelines. According to the American Society of Clinical Oncology and College of American Pathologists guidelines: ER/PR positivity was defined as ≥1% nuclear staining (20), HER2 status was categorized as negative [immunohistochemistry (IHC) 0/1+], equivocal [IHC 2+, requiring FISH validation] (12), or positive (IHC 3+), and Ki-67 expression was stratified as low (<30% labeled nuclei) or high (≥30%) (21).

According to the updated guidelines from the American Society of Clinical Oncology (ASCO) and the College of American Pathologists (CAP), the classification of HER2 expression in breast cancer has been refined. A new category, termed HER2-low, has been introduced to distinguish tumors previously grouped within the HER2-negative cohort. HER2-low tumors are defined as those with IHC 1+ or IHC 2+ expression and negative FISH results (13). Based on this revised version, our study adopted a three-tier classification of HER2 expression: HER2-zero (IHC 0, ineligible for conventional HER2-targeted therapy), HER2-low (potential candidates for novel HER2-targeted agents), and HER2-positive (IHC 3+ or IHC 2+/FISH-positive, eligible for traditional HER2-targeted medications) (11).

Based on St. Gallen International Consensus criteria (22), invasive ductal carcinomas were classified into four molecular subtypes: Luminal A (ER+ and/or PR+, HER2−, Ki-67< 14%), Luminal B (ER+ and/or PR+ with HER2+ or HER2− but Ki-67 ≥14%), HER2-enriched (ER−, PR− and HER2+), and TN (ER−, PR− and HER2−).

### Statistical analysis

All statistical analyses were performed using SPSS version 22.0 for Windows (IBM, USA) and MadCalc version 20.0 (MedCalc Software, Belgium). Interobserver agreement was evaluated using the intraclass correlation coefficient (ICC), with values ranging from 0.81 to 1.00 indicating excellent consistency (23). If the quantitative parameters measured by the two observers showed good consistency, the data from the more experienced observer would be used for subsequent statistical analysis. Based on breast cancer molecular subtypes, patients were divided into four groups. The baseline clinical and MRI characteristics of the four groups, including quantitative T_d_-dMRI metrics—ADC_50ms_, ADC_30ms_, ADC_25Hz_, ADC_50Hz_, Cellularity, D_ex_, Cell diameter, D_in_, f_in_ and τ_in_—were compared using the analysis of variance (ANOVA) or the Kruskal-Wallis H test for continuous variables and the χ² test or Fisher exact test for categorical variables. When the ANOVA or Kruskal-Wallis H test indicated significant intergroup differences (P< 0.05), Bonferroni-adjusted pairwise comparisons were conducted to identify significantly different groups. Pairwise comparisons were also conducted for categorical variables showing significant differences.

Patients were further categorized into subtypes based on ER (+/−), PR (+/−), HER-2 expression (+/−), and Ki-67 proliferation index (low Ki-67< 30% and high Ki-67 ≥ 30%). Following verification of normal distribution and homogeneity of variance, quantitative parameters from T_d_-dMRI were compared between subgroups using independent samples t-tests, while nonparametric Mann-Whitney U test was applied to non-normally distributed or heterogeneity of variance data. The diagnostic performance of quantitative parameters was evaluated using receiver operating characteristic (ROC) curve analysis, with inter-parameter comparisons of the area under the curve (AUC) conducted via DeLong’s test. A two-tailed threshold of *P* < 0.05 indicated statistical significance.

## Results

### Participant characteristics

The final cohort comprised 71 female patients (mean age: 51.3 ± 10.2 years; range, 23–76) with 71 histologically confirmed breast invasive ductal carcinoma (**Figure 1**). The comprehensive baseline clinical and MRI findings for all participants are presented in **Table 1**.

**Table 1:** The baseline characteristics of participants.

Characteristic	Total (n=71)	Molecular subtypes				χ^2^	*P*-value
		Luminal A (n=5)	Luminal B (n=41)	HER2-enriched (n=17)	TN (n=8)		
Age (y) *	51.3 ± 10.2	51.0 ± 14.5	51.0 ± 11.0	53.0 (16.0)	55.5 ± 6.8	/	0.590 ^†^
Menstrual status						2.465	0.482 ^‡^
Premenopausal	35 (49%)	3 (9%)	22 (63%)	8 (23%)	2 (6%)		
Postmenopausal	36 (51%)	2 (6%)	19 (53%)	9 (25%)	6 (16%)		
Breast lesion laterality						2.267	0.519 ^‡^
Left	37 (52%)	2 (5%)	19 (51%)	11 (30%)	5 (14%)		
Right	34 (48%)	3 (9%)	22 (64%)	6 (18%)	3 (9%)		
Reproductive history						0.874	>0.999 ^‡^
Parous	65 (92%)	5 (8%)	37 (57%)	15 (23%)	8 (12%)		
Nulliparous	6 (8%)	0 (0%)	4 (67%)	2 (33%)	0 (0%)		
Breastfeeding history						4.128	0.938 ^‡^
No breastfeeding	12 (17%)	1 (8%)	7 (59%)	3 (25%)	1 (8%)		
Breastfeeding< 6 months	18 (25%)	1 (6%)	9 (50%)	6 (33%)	2 (11%)		
Breastfeeding 6–12 months	33 (47%)	3 (9%)	18 (55%)	7 (21%)	5 (15%)		
Breastfeeding > 12 months	8 (11%)	0 (0%)	7 (88%)	1 (12%)	0 (0%)		
Histological grade (HG)						10.889	0.008 ^‡^
HG 1-2	43 (61%)	5 (12%)	29 (67%)	6 (14%)	3 (7%)		
HG 3	28 (39%)	0 (0%)	12 (43%)	11 (39%)	5 (18%)		
Lymph node metastasis						7.945	0.040 ^‡^
Positive	41 (58%)	0 (0%)	27 (66%)	10 (24%)	4 (10%)		
Negative	30 (42%)	5 (17%)	14 (47%)	7 (23%)	4 (13%)		
TNM staging							
T stage						15.296	0.034 ^‡^
T1	12 (17%)	4 (33%)	7 (58%)	1 (9%)	0 (0%)		
T2	39 (55%)	0 (0%)	22 (56%)	12 (31%)	5 (13%)		
T3	13 (18%)	1 (8%)	8 (61%)	3 (23%)	1 (8%)		
T4	7 (10%)	0 (0%)	4 (57%)	1 (14%)	2 (29%)		
N stage						13.254	0.084 ^‡^
N0	30 (42%)	5 (17%)	14 (47%)	7 (23%)	4 (13%)		
N1	24 (34%)	0 (0%)	18 (75%)	5 (21%)	1 (4%)		
N2	8 (11%)	0 (0%)	6 (75%)	0 (0%)	2 (25%)		
N3	9 (13%)	0 (0%)	4 (44%)	4 (44%)	1 (12%)		
M stage						0.957	>0.999 ^‡^
M0	68 (96%)	5 (7%)	39 (57%)	16 (24%)	8 (12%)		
M1	3 (4%)	0 (0%)	2 (67%)	1 (33%)	0 (0%)		
Time-signal intensity curve type						8.005	0.183 ^‡^
Persistent	3 (4%)	2 (67%)	1 (33%)	0 (0%)	0 (0%)		
Plateau	26 (37%)	1 (4%)	16 (62%)	6 (23%)	3 (11%)		
Washout	42 (59%)	2 (5%)	24 (57%)	11 (26%)	5 (12%)		
BI-RADS						2.586	0.392 ^‡^
Negative (grades 1, 2, 3)	4 (6%)	1 (25%)	2 (50%)	1 (25%)	0 (0%)		
Positive (grades 4 and 5)	67 (94%)	4 (6%)	39 (58%)	16 (24%)	8 (12%)		
Largest diameter of lesion (mm) *	29.1 ± 9.4	22.3 ± 6.2	30.1 ± 10.5	28.6 ± 7.4	29.1 ± 8.7	/	0.413 ^†^
Shape						4.029	0.254 ^‡^
Oval or round	49 (69%)	5 (10%)	29 (59%)	9 (19%)	6 (12%)		
Irregular	22 (31%)	0 (0%)	12 (55%)	8 (36%)	2 (9%)		
Margin						2.931	0.402 ^‡^
Circumscribed	40 (56%)	4 (10%)	24 (60%)	7 (18%)	5 (12%)		
Not circumscribed	31 (44%)	1 (3%)	17 (55%)	10 (32%)	3 (10%)		
Internal enhancement						7.385	0.558 ^‡^
Homogeneous	19 (27%)	3 (16%)	10 (53%)	5 (26%)	1 (5%)		
Heterogeneous	38 (54%)	2 (5%)	24 (63%)	8 (21%)	4 (11%)		
Rim	11 (15%)	0 (0%)	5 (46%)	4 (36%)	2 (18%)		
Others	3 (4%)	0 (0%)	2 (67%)	0 (%)	1 (33%)		

Unless otherwise indicated, data are number of patients, and data in parentheses are percentages.

BI-RADS, Breast Imaging Reporting and Data System; TN, triple-negative.

*Data are presented as mean ± SD (normally distributed) or median [interquartile range] (non-normally distributed).

The P value ^†^ was calculated using the ANOVA or Kruskal-Wallis H test.

The P value ^‡^ was calculated using the χ^2^ test or Fisher exact test.

The data in the ‘Total’ and ‘Molecular Subtypes’ columns, formatted as XX (XX%), denoted the number of cases (percentage proportion).

The distributions of age, menstrual status, breast lesion laterality, reproductive history, breastfeeding history, N and M stages of TNM stages, time-signal intensity curve type, BI-RADS results, largest diameter of lesion, shape, margin and internal enhancement pattern did not differ among the four molecular subtypes of breast cancer (P = 0.590, P = 0.482, P = 0.519, P > 0.999, P = 0.938, P = 0.084, P = 1.000, P = 0.183, P = 0.392, P = 0.413, P = 0.254, P = 0.402 and P = 0.558, respectively). However, the histological grade, lymph node metastasis status, and T stage of four subtypes demonstrated significant inter-subtype differences (P = 0.008, 0.040, and 0.034, respectively). *Post hoc* pairwise comparisons with Bonferroni correction revealed that lymph node metastasis differed significantly between the Luminal A and Luminal B subtypes (adjusted P = 0.005), while T stage differed significantly between Luminal A and HER2-enriched (adjusted P = 0.002), as well as between Luminal A and TN subtypes (adjusted P = 0.004).

### Inter-observer assessment

The inter-observer agreement between the 2 radiologists for parameter measurements were excellent, with all ICCs exceeding 0.8 for quantitative metrics of T_d_-dMRI. Consequently, the average values from both observers were used for subsequent statistical analyses.

### Association of T_d_-dMRI quantitative metrics with molecular status

#### Comparison among the four molecular subtypes

**Figure 2:**
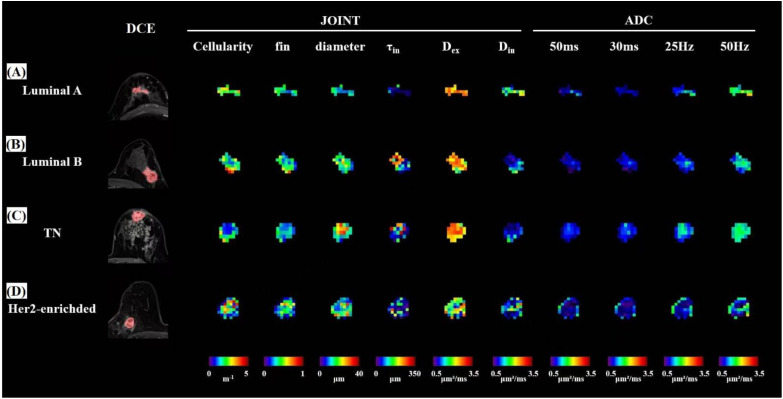
Diffusivity MRI maps in four participants with different molecular subtypes using pulsed gradient spin-echo (PGSE) and oscillating gradient spin-echo (OGSE) sequences, including cellularity, intracellular volume fraction (f_in_), cell diameter, intracellular water residence time (τ_in_), extracellular and intracellular diffusivity (D_ex_, D_in_), and the four ADC maps (ADC_50ms_, ADC_30ms_, ADC_25Hz_, and ADC_50Hz_). The bottom of figure shows the corresponding specific values. **(A)** A 45-year-old female with Luminal A breast cancer; **(B)** a 50-year-old female with Luminal B breast cancer; **(C)** a 49-year-old female with triple-negative (TN) breast cancer; **(D)** a 39-year-old female with HER2-enriched breast cancer.

**Figure 3:**
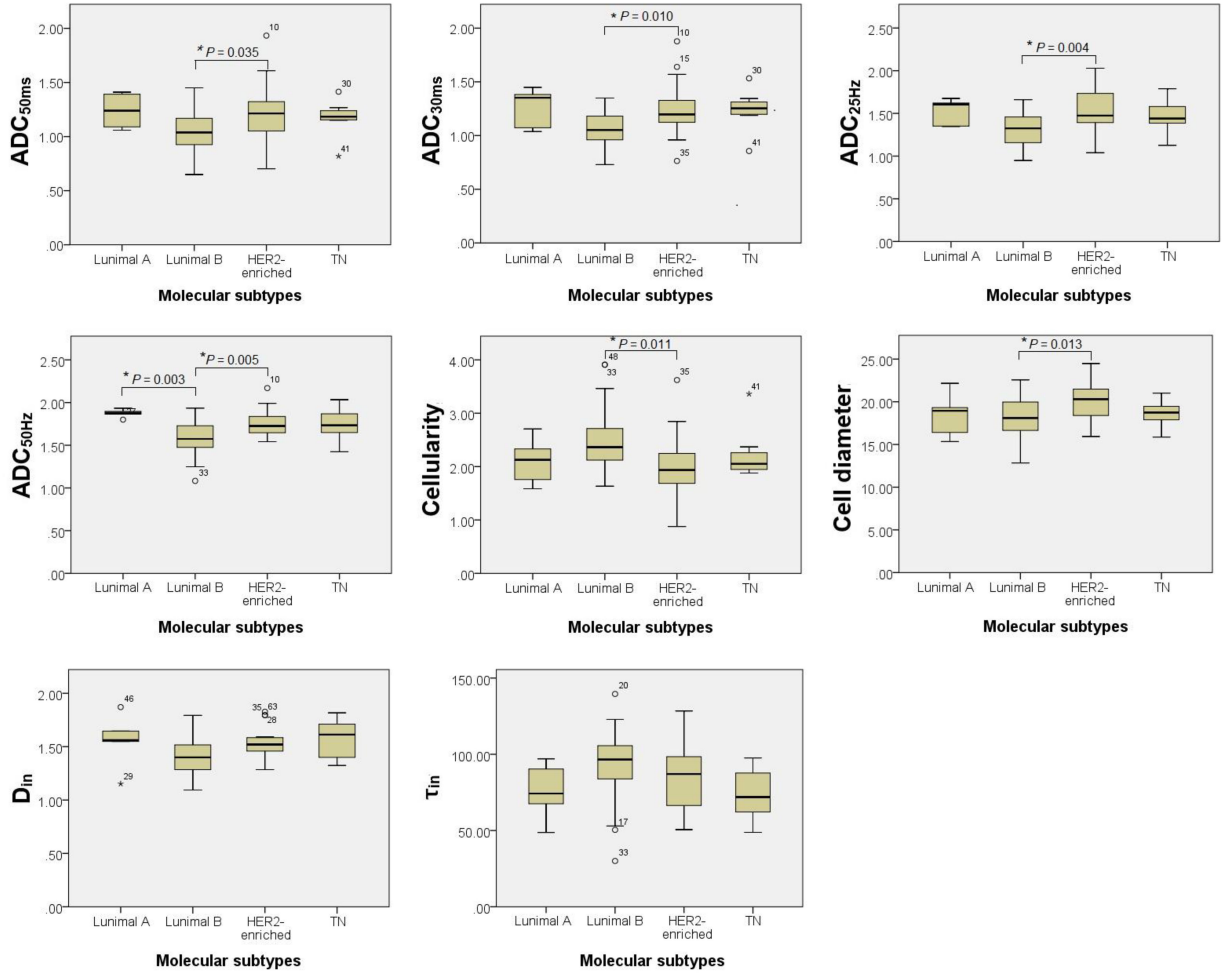
*Post-hoc* pairwise comparisons with Bonferroni correction of T_d_-dMRI quantitative metrics among four molecular subtypes. *means adjusted *P* < 0.05.

ADC_50ms_, ADC_30ms_, ADC_25Hz_, ADC_50Hz_, Cellularity, Diameter, D_in_ and τ_in_ values had significantly differences among four molecular subtypes of breast cancer (**Table 2**, **Figure 2**). Subsequent Bonferroni-adjusted *post-hoc* analyses demonstrated that the ADC_50Hz_ values in Luminal A breast cancer were significantly higher than those in Luminal B subtype (adjusted *P* = 0.003). Additionally, the HER2-enriched subtype exhibited significantly higher ADC_50ms_, ADC_30ms_, ADC_25Hz_, ADC_50Hz_, and Cell diameter values compared to Luminal B (adjusted *P* = 0.035, 0.010, 0.004, 0.005, 0.013, respectively). Conversely, the HER2-enriched subtype showed significantly lower Cellularity values than Luminal B (adjusted *P* = 0.011), as shown in **Figure 3**.

**Table 2:** Comparison of T_d_-dMRI quantitative metrics among four molecular subtypes of breast cancer.

T_d_-dMRI metrics	Molecular subtypes				F/H value	*P* value
	Luminal A (n=5)	Luminal B (n=41)	HER2-enriched (n=17)	TN (n=8)		
ADC-50ms (μm²/ms)	1.24 ± 0.16	1.04 ± 0.19	1.22 ± 0.29	1.17 ± 0.17	3.647	**0.017** ^†^
ADC-30ms (μm²/ms)	1.26 ± 0.19	1.07 ± 0.16	1.25 ± 0.27	1.24 ± 0.19	5.058	**0.003** ^†^
ADC-25Hz (μm²/ms)	1.52 ± 0.16	1.31 ± 0.18	1.52 ± 0.26	1.47 ± 0.20	5.386	**0.002** ^†^
ADC-50Hz (μm²/ms)	1.87 ± 0.05	1.57 ± 0.18	1.75 ± 0.16	1.74 ± 0.20	8.123	**<0.001** ^†^
Cellularity	2.10 ± 0.45	2.36 (0.71)	2.02 ± 0.62	2.05 (0.38)	11.610	**0.009** ^‡^
D_ex_ (μm²/ms)	2.48 ± 0.11	2.31 (0.16)	2.31 ± 0.13	2.33 (0.34)	7.430	0.059 ^‡^
Cell diameter (μm)	18.43 ± 2.67	18.13 ± 2.46	20.38 ± 2.64	18.63 ± 1.55	3.458	**0.021** ^†^
D_in_ (μm²/ms)	1.56 ± 0.26	1.42 ± 0.19	1.53 ± 0.16	1.57 ± 0.18	2.871	**0.043** ^†^
f_in_	0.37 ± 0.06	0.42 ± 0.06	0.38 ± 0.09	0.36 (0.08)	6.196	0.102 ^‡^
τ_in_ (ms)	75.59 ± 19.12	92.78 ± 21.22	84.12 ± 20.18	73.76 ± 16.85	2.839	**0.044** ^†^

Data are presented as mean ± SD (normally distributed) or median [interquartile range] (non-normally distributed).

The P value in bold indicates that P< 0.05.

The P value ^†^ was calculated using the ANOVA test, and the P value ^‡^ was calculated using Kruskal-Wallis H test and adjusted by Bonferroni.

**Figure 4:**
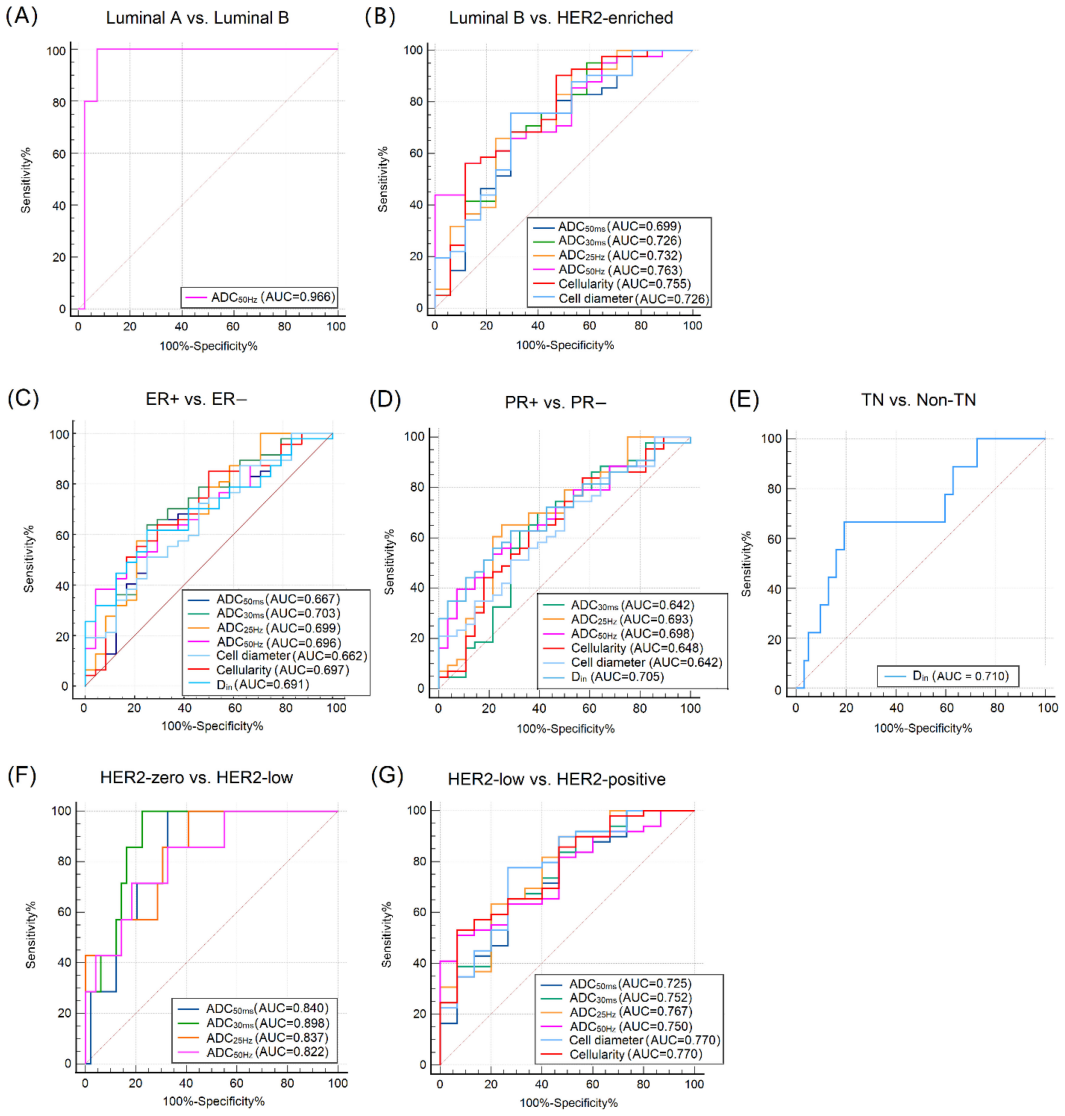
ROC curves of T_d_-dMRI metrics for differentiating subtypes of breast cancer. **(A)** Luminal A vs. Luminal B, **(B)** Luminal B vs. HER2-enriched, **(C)** ER+ vs. ER−, **(D)** PR+ vs. PR−, **(E)** TN vs. Non-TN, **(F)** HER2-zero vs. HER2-low, **(G)** HER2-low vs. HER2-positive breast cancer.

Analysis of these statistically significant parameters revealed diagnostic performance with area under the curve (AUC) values ranging from 0.699 to 0.966. Notably, ADC_50Hz_ demonstrated excellent diagnostic efficacy in differentiating Luminal A from Luminal B subtypes (AUC = 0.966). For distinguishing Luminal B and HER2-enriched subtypes, ADC_50Hz_ showed the optimal diagnostic performance (AUC = 0.763) (**Table 3**, **Figure 4**).

**Table 3:** Diagnostic performance of T_d_-dMRI metrics in differentiating molecular subtypes of breast cancer.

Subtype comparison	Parameters	AUC (95% CI)	Adjusted *P* value*	Cut-off	Sensitivity(%)	Specificity(%)	Accuracy(%)	PPV (%)	NPV(%)
Luminal A vs. Luminal B	ADC_50Hz_ (μm²/ms)	0.966 (0.865-0.997)	0.003	1.78	100.0 (5/5)	92.7 (38/41)	93.5 (43/46)	62.5 (5/8)	100.0 (38/38)
Luminal B vs. HER2-enriched	ADC_50ms_ (μm²/ms)	0.699 (0.564-0.812)	0.035	1.11	68.3 (28/41)	70.6 (12/17)	70.0 (40/58)	84.8 (28/33)	48.0 (12/25)
	ADC_30ms_ (μm²/ms)	0.726 (0.593-0.835)	0.010	1.15	68.3 (28/41)	70.6 (12/17)	70.0 (40/58)	84.8 (28/33)	48.0 (12/25)
	ADC_25Hz_ (μm²/ms)	0.732 (0.599-0.840)	0.004	1.39	65.9 (27/41)	76.5 (13/17)	70.0 (40/58)	87.1 (27/31)	48.1 (13/27)
	ADC_50Hz_ (μm²/ms)	0.763 (0.633-0.865)	0.005	1.59	56.1 (23/41)	88.2 (15/17)	65.5 (38/58)	92.0 (23/25)	45.5 (15/33)
	Cellularity	0.755 (0.624-0.858)	0.011	2.34	56.1 (23/41)	88.2 (15/17)	65.5 (38/58)	92.0 (23/25)	45.5 (15/33)
	Cell diameter (μm)	0.726 (0.593-0.835)	0.013	19.97	75.6 (31/41)	70.6 (12/17)	74.1 (43/58)	86.1 (31/36)	54.5 (12/22)

Adjusted *P* value refers to P values obtained through Bonferroni-corrected *post-hoc* pairwise comparisons.

PPV, positive predictive value; NPV, negative predictive value.

#### Comparison of different subtypes based on ER, PR, and Ki-67 expression and TN status

**Figure 5:**
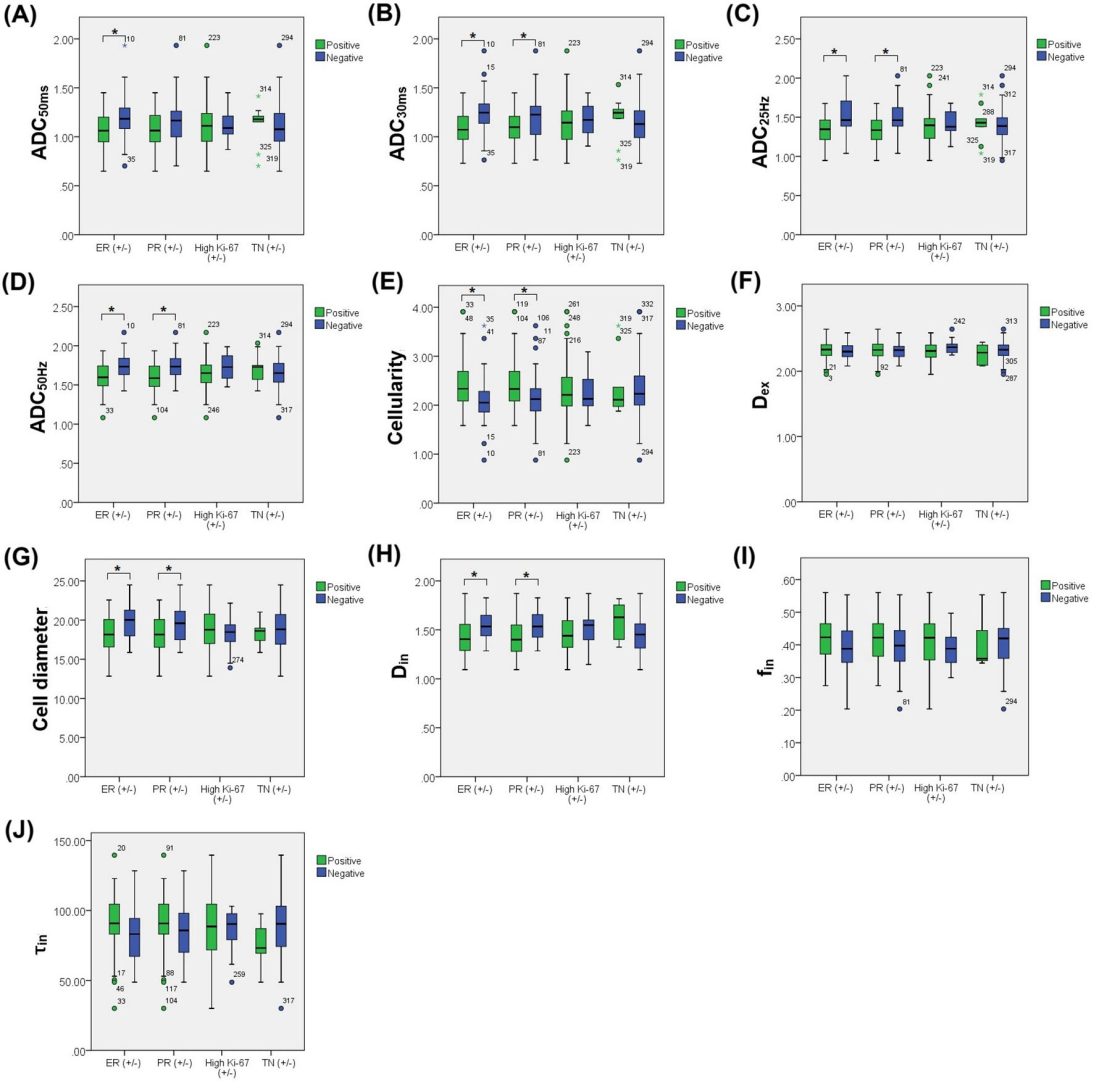
Box and whisker plots show the comparisons of T_d_-dMRI metrics between different ER, PR, and Ki-67 expression and TN status, including ADC_50ms_ **(A)**, ADC_30ms_ **(B)**, ADC_25Hz_ **(C)**, ADC_50Hz_ **(D)**, Cellularity **(E)**, D_ex_ **(F)**, Cell diameter **(G)**, D_in_ **(H)**, f_in_ **(I)** and τ_in_ **(J)**. The *P* < 0.05 was denoted in the plots using *. Whiskers denote the range in each group, dots represent individual data points, boxes indicate the SD, and midlines are the median.

Several quantitative T_d_-dMRI parameters demonstrated significant variation across molecular subtypes defined by ER, PR, and Ki-67 expression and TN status. ER− and PR− tumors exhibited significantly higher ADC values, cell diameters and D_in_ compared to their positive counterparts. These tumors also showed significantly lower cellularity. Notably, TN breast cancers demonstrated significantly higher D_in_ values compared to non-TN tumors. In contrast, no statistically significant differences in any T_d_-dMRI metrics were observed between high and low Ki-67 expression groups (all *P* > 0.05). Complete comparative results are detailed in **Figure 5**, and the comparison results with statistical differences are detailed in **Table 4**.

**Table 4:** Comparison of T_d_-dMRI metrics between different subtypes.

Subtype comparison	Parameters	Status		t/Z value	*P* value
		Positive	Negative		
ER+ vs. ER−	ADC_50ms_ (μm²/ms)	1.07 ± 0.19	1.20 ± 0.26	2.501	**0.015**
	ADC_30ms_ (μm²/ms)	1.09 ± 0.17	1.25 ± 0.25	3.194	**0.002**
	ADC_25Hz_ (μm²/ms)	1.34 ± 0.18	1.51 ± 0.24	3.262	**0.002**
	ADC_50Hz_ (μm²/ms)	1.61 ± 0.19	1.75 ± 0.17	3.065	**0.003**
	Cellularity	2.34 (0.65)	2.05 (0.45)	-2.699	**0.007** ^†^
	Cell diameter (μm)	18.21 ± 2.44	19.81 ± 2.51	2.590	**0.012**
	D_in_ (μm²/ms)	1.43 ± 0.20	1.55 ± 0.16	2.615	**0.011**
PR+ vs. PR−	ADC_30ms_ (μm²/ms)	1.10 ± 0.17	1.21 ± 0.25	2.293	**0.025**
	ADC_25Hz_ (μm²/ms)	1.34 ± 0.18	1.49 ± 0.24	3.042	**0.003**
	ADC_50Hz_ (μm²/ms)	1.60 ± 0.19	1.74 ± 0.17	3.135	**0.003**
	Cellularity	2.33 (0.65)	2.12 (0.46)	-2.094	**0.036**
	Cell diameter (μm)	18.19 ± 2.50	19.61 ± 2.45	2.366	**0.021**
	D_in_ (μm²/ms)	1.42 ± 0.20	1.55 ± 0.16	2.962	**0.004**
TN vs. Non-TN	D_in_ (μm²/ms)	1.60 ± 0.18	1.45 ± 0.19	-2.154	**0.035**

The *P* value ^†^ was calculated using the Mann-Whitney U test, and other P values were calculated using independent samples t-test. The P value in bold indicates that P < 0.05.

ROC analysis demonstrated that ADC values, cellularity, cell diameter, and D_in_ yielded moderate diagnostic performance for differentiating ER and PR expression. Among these parameters, ADC_30ms_ achieved the highest AUC for ER status (AUC = 0.703), while D_in_ provided the highest AUC for PR status (AUC = 0.705). Additionally, D_in_ showed moderate utility in distinguishing TN tumors from non-TN tumors (AUC = 0.710). The diagnostic performance metrics for all relevant parameters are summarized in **Table 5** and **Figure 4**.

**Table 5:** ROC analysis for subtype classification.

Subtype comparison	Parameters	AUC (95% CI)	Cut-off	Sensitivity(%)	Specificity(%)	Accuracy(%)	PPV(%)	NPV(%)
ER+ vs. ER−	ADC_50ms_ (μm²/ms)	0.667 (0.545-0.774)	1.11	63.8 (30/47)	75.0 (18/24)	67.6 (48/71)	83.3 (30/36)	51.4 (18/35)
	ADC_30ms_ (μm²/ms)	0.703 (0.583-0.806)	1.15	63.8 (30/47)	75.0 (18/24)	67.6 (48/71)	83.3 (30/36)	51.4 (18/35)
	ADC_25Hz_ (μm²/ms)	0.699 (0.578-0.802)	1.39	61.7(29/47)	75.0 (18/24)	66.2 (47/71)	82.9 (29/35)	50.0 (18/36)
	ADC_50Hz_ (μm²/ms)	0.696 (0.575-0.800)	1.54	38.3 (18/47)	95.8 (23/24)	57.7 (41/71)	94.7 (18/19)	44.2 (23/52)
	Cellularity	0.697 (0.576-0.800)	1.99	85.1 (40/47)	50.0 (12/24)	73.2 (52/71)	76.9 (40/52)	63.2 (12/19)
	Cell diameter (μm)	0.662 (0.540-0.770)	19.76	72.3 (34/47)	54.2 (13/24)	66.2 (47/71)	75.6 (34/45)	50.0 (13/26)
	D_in_ (μm²/ms)	0.691 (0.570-0.795)	1.45	61.7 (29/47)	75.0 (18/24)	66.2 (47/71)	82.9 (29/35)	50.0 (18/36)
PR+ vs. PR−	ADC_30ms_ (μm²/ms)	0.642 (0.519-0.752)	1.15	62.8 (27/43)	67.9 (19/28)	64.8 (46/71)	75.0 (27/36)	67.9 (19/28)
	ADC_25Hz_ (μm²/ms)	0.693 (0.572-0.797)	1.39	65.1 (28/43)	75.0 (21/28)	69.0 (49/71)	80.0 (28/35)	58.3 (21/36)
	ADC_50Hz_ (μm²/ms)	0.698 (0.577-0.801)	1.65	62.8 (27/43)	71.4 (20/28)	66.2 (47/71)	77.1 (27/35)	71.4 (20/28)
	Cellularity	0.648 (0.525-0.758)	2.18	62.8 (27/43)	64.3 (18/28)	63.4 (45/71)	73.0 (27/37)	52.9 (18/34)
	Cell diameter (μm)	0.642 (0.519-0.752)	18.14	51.1 (22/43)	71.4 (20/28)	59.2 (42/71)	73.3 (22/30)	71.4 (20/28)
	D_in_ (μm²/ms)	0.705 (0.585-0.807)	1.41	55.8 (24/43)	78.6 (22/28)	64.8 (46/71)	80.0 (24/30)	78.6 (22/28)
TN vs. Non-TN	D_in_ (μm²/ms)	0.710 (0.590-0.811)	1.59	66.7 (6/9)	80.7 (50/62)	78.9 (56/71)	33.3 (6/18)	94.3 (50/53)

PPV, positive predictive value; NPV, negative predictive value.

#### Comparison among HER2-zero, HER2-low, and HER2-positive breast cancer subtypes

**Figure 6:**
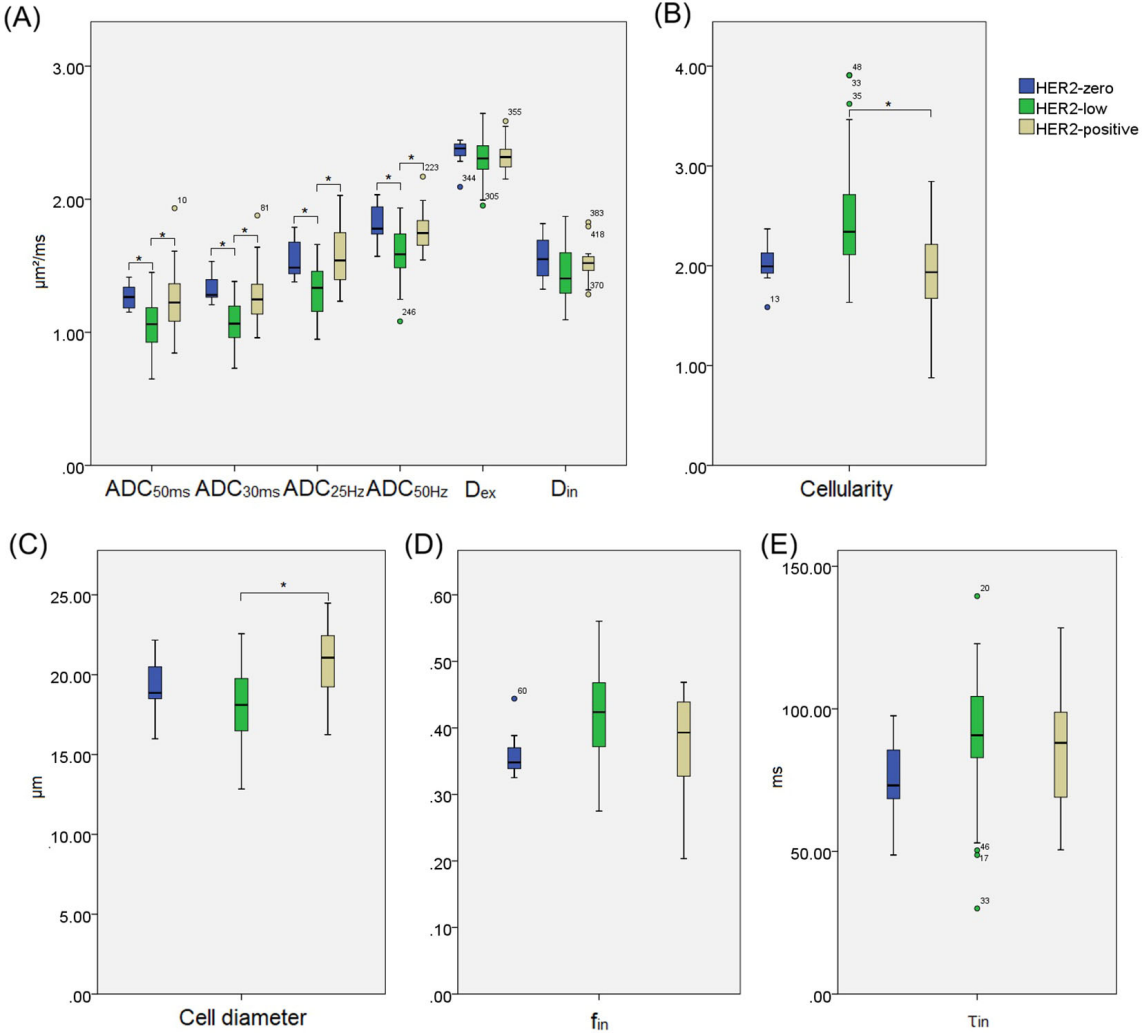
Box and whisker plots show the comparisons of T_d_-dMRI metrics HER2-zero, HER2-low, and HER2-positive breast cancer, including ADC_50ms_, ADC_30ms_, ADC_25Hz_, ADC_50Hz_ **(A)**, Cellularity **(B)**, D_ex_ **(A)**, Cell diameter **(C)**, D_in_ **(A)**, f_in_ **(D)** and τ_in_ **(E)**. The *P* < 0.05 was denoted in the plots using *. Whiskers denote the range in each group, dots represent individual data points, boxes indicate the SD, and midlines are the median.

Quantitative analysis revealed that several T_d_-dMRI-derived parameters effectively differentiated HER2 expression subtypes (**Figure 6**). Compared with HER2-low tumors, HER2-zero lesions demonstrated significantly higher ADC values across multiple diffusion time scales (ADC_50ms_, ADC_30ms_, ADC_25Hz_, ADC_50Hz_), while no differences were observed in microstructural parameters. In contrast, HER2-positive tumors exhibited significantly higher ADC values, reduced cellularity, and larger cell diameters compared with HER2-low tumors.

For distinguishing HER2-zero from HER2-low tumors, all ADC metrics showed strong performance. ADC_30ms_ yielded the highest AUC (0.898, 95% CI: 0.788–0.963), with 100.0% sensitivity and 77.6% specificity at a cut-off of 1.20 μm²/ms. Other ADC parameters (ADC_50ms_, ADC_25Hz_, ADC_50Hz_) also demonstrated high diagnostic value (AUCs > 0.82). When comparing HER2-low to HER2-positive tumors, Cell diameter and Cellularity each achieved the best performance (AUC = 0.770), followed by ADC_25Hz_, ADC_30ms_, ADC_50Hz_ and ADC_50ms_ (AUCs 0.725–0.767). A cell diameter threshold of 19.97 μm provided 77.6% sensitivity and 73.3% specificity, while a cellularity threshold of 2.34 demonstrated high specificity (93.3%) and moderate sensitivity (53.1%) (**Table 6**, **Figure 4**).

**Table 6:** ROC analysis of T_d_-dMRI metrics for differentiating HER2-zero, HER2-low, and HER2-positive breast cancer.

Subtype comparison	Parameters	*P* value	AUC (95% CI)	Cut-off	Sensitivity (%)	Specificity (%)	Accuracy (%)	PPV (%)	NPV (%)
HER2-zero vs. HER2-low	ADC_50ms_ (μm²/ms)	0.010	0.840 (0.717-0.924)	1.13	100.0 (7/7)	67.4 (33/49)	71.4 (40/56)	30.4 (7/23)	100.0 (33/33)
	ADC_30ms_ (μm²/ms)	0.001	0.898 (0.788-0.963)	1.20	100.0 (7/7)	77.6 (38/49)	80.4 (45/56)	38.9 (7/18)	100.0 (38/38)
	ADC_25Hz_ (μm²/ms)	0.004	0.837 (0.714-0.922)	1.38	100.0 (7/7)	59.2 (29/49)	64.3 (36/56)	25.9 (7/27)	100.0 (29/29)
	ADC_50Hz_ (μm²/ms)	0.003	0.822 (0.697-0.911)	1.74	71.4 (5/7)	81.6 (40/49)	80.4 (45/56)	35.7 (5/14)	95.2 (40/42)
HER2-low vs. HER2-positive	ADC_50ms_ (μm²/ms)	0.001	0.725 (0.599-0.829)	1.11	65.3 (32/49)	73.3 (11/15)	67.2 (43/64)	88.9 (32/36)	39.3 (11/28)
	ADC_30ms_ (μm²/ms)	<0.001	0.752 (0.629-0.852)	1.12	59.2 (29/49)	80.0 (12/15)	64.1 (41/64)	90.6 (29/32)	37.5 (12/32)
	ADC_25Hz_ (μm²/ms)	<0.001	0.767 (0.645-0.864)	1.39	63.3 (31/49)	80.0 (12/15)	67.2 (43/64)	91.2 (31/34)	40.0 (12/30)
	ADC_50Hz_ (μm²/ms)	0.002	0.750 (0.626-0.850)	1.59	51.0 (25/49)	93.3 (14/15)	60.9 (39/64)	96.2 (25/26)	36.8 (14/38)
	Diameter (μm)	<0.001	0.770 (0.648-0.866)	19.97	77.6 (38/49)	73.3 (11/15)	76.6 (49/64)	90.5 (38/42)	50.0 (11/22)
	Cellarity	0.004^†^	0.770 (0.648-0.866)	2.34	53.1 (26/49)	93.3 (14/15)	62.5 (40/64)	96.3 (26/27)	37.8 (14/37)

The *P* value^†^ was obtained by *post-hoc* pairwise comparison after being corrected by Bonferroni.

PPV, positive predictive value; NPV, negative predictive value.

## Discussion

This study demonstrates that T_d_-dMRI metrics—particularly ADC values obtained across different diffusion times and oscillation frequencies—along with microstructural parameters such as cellularity, cell diameter, and D_in_, possess significant discriminatory potential for non-invasively characterizing breast cancer molecular subtypes and receptor status, including the recently emphasized HER2-low category. Notably, ADC_30ms_ achieved the highest diagnostic performance for distinguishing HER2-zero from HER2-low tumors (AUC = 0.898), while D_in_, cellularity, and cell diameter were particularly effective in differentiating HER2-low from HER2-positive tumors, highlighting the strength of T_d_-dMRI in addressing this emerging three-tier classification of HER2 expression. Additionally, ADC values and D_in_ showed consistent performance in differentiating ER, PR, and TN breast cancer status. Furthermore, no statistically significant differences in any T_d_-dMRI metrics were found between high and low Ki-67 expression groups.

Compared to prior studies utilizing mono-exponential models or conventional DWI-derived ADC values—which have reported limited specificity in distinguishing molecular subtypes (24, 25)—our findings underscore the added value of T_d_-dMRI. By varying diffusion times and oscillation frequencies, T_d_-dMRI enables more nuanced probing of water diffusion, potentially enhancing sensitivity to microstructural differences. Furthermore, evidence from prior *in vitro* and animal studies indicated that T_d_-dMRI can capture quantitative characterization of microstructure at length scales that are clinically relevant (e.g, 10 – 20 μm corresponding to many cell sizes), such as cell size, membrane permeability, and extracellular matrix organization (3, 7, 26). These foundational findings supported the biological plausibility of applying T_d_-dMRI to quantify tumor microstructure *in vivo*. Consistent with previous reports that conventional ADC values tend to be lower in ER+ tumors and higher in TN and HER2-enriched subtypes (25, 27), our results not only indicated these trends but also revealed improved diagnostic granularity using T_d_-dMRI. Unlike Liu et al. and Chang et al. studies (28–30), which reported limited ability of conventional ADC values to distinguish PR status, our study demonstrated that T_d_-dMRI parameters, particularly D_in_, can effectively differentiate PR+ from PR− breast cancers, potentially providing a novel noninvasive tool for predicting PR status. Notably, we suggested that ADC metrics obtained from specific diffusion conditions (e.g., ADC_30ms_ and ADC_50ms_) outperformed conventional parameters in differentiating HER2-low from HER2-zero tumors, a recent subtype distinction not addressed in earlier DWI studies (11, 13). In addition, we indicated the microstructural parameters, such as cellularity and cell diameter, could also effectively distinguish HER2-low from HER2-positive tumors, further supporting HER2-low as a biologically and therapeutically distinct subtype (14). The findings suggested that T_d_-dMRI might have the potential to capture microstructural differences that could not be detected by conventional diffusion imaging (4, 10).This study observed significant differences in multiple diffusion and microstructural features derived from T_d_-dMRI among different breast cancer subtypes, which might be reasonably explained by underlying pathophysiological mechanisms. Among the four molecular subtypes, Luminal A tumors exhibited significantly higher ADC values compared to the relatively more aggressive Luminal B subtype. This difference might be due to the generally favorable prognosis and lower proliferative activity of Luminal A tumors, whereas Luminal B cancers typically displayed higher cellular density and reduced extracellular space, restricting water diffusion and consequently lowering ADC values. Similar results were observed in studies by Montemezzi et al. and Martincich et al. (31, 32). In addition, our analysis of clinical characteristics also revealed that Luminal A tumors had a significantly lower rate of lymph node metastasis than Luminal B and were more likely to present with an earlier T stage compared with HER2-eriched and TN subtypes. These trends aligned with the generally less aggressive biological behavior reported for Luminal A breast cancers and provided supportive context for the imaging-based associations observed in this study. Conversely, HER2-enriched breast cancers demonstrated higher ADC values (ADC_50ms_, ADC_30ms_, ADC_25Hz_, ADC_50Hz_), larger cell diameters, and lower cellularity compared to Luminal B, which were consistent with prior researches by Kim et al. and Wang et al. (9, 33). This pattern was likely attributed to the rapid proliferation and high metabolic activity characteristic of HER2-enriched tumors, which were more susceptible to intratumoral necrosis and cystic degeneration. Although ROI delineation in this study had avoided visibly identifiable cystic or necrotic regions, small areas of necrosis or cystic degeneration that were not easily detectable might still be present. These changes resulted in relatively less compact cellular architecture, thereby facilitating increased water diffusivity.

Our study also revealed that ER− and PR− tumors, including TN breast cancers, demonstrated significantly higher ADC values, larger cell diameters, and increased D_in_, along with reduced cellularity. These findings might be attributed to the following fundamental differences in tumor biology and pathophysiological processes associated with hormone receptor status. Firstly, ER+ and PR+ tumors, typically well-differentiated and associated with the luminal subtypes, exhibited slower proliferation and responded to hormonal regulation of cell growth and apoptosis (34, 35). These features resulted in tightly packed, smaller cells with limited extracellular space, leading to physically restricted water diffusion and lower ADC and D_in_ values. In contrast, ER− and PR− tumors, especially TN subtypes, were high-grade, poorly differentiated, and highly proliferative, with disorganized architecture, larger pleomorphic cells. The absence of hormone receptor signaling led to unregulated growth that often outpaced the development of supportive stroma and vasculature, contributing to intratumoral hypoxia. This hypoxic environment promoted necrosis and cystic degeneration, which expanded extracellular space and reduced viable cell density, resulting in decreased cellularity and increased water diffusivity (35), reflected by elevated ADC and D_in_ values. Secondly, the elevated D_in_ values observed in ER− and PR− tumors might also be attributed to subcellular alterations characteristic of aggressive phenotypes. These included compromised membrane integrity, decreased cytoplasmic viscosity, and dysregulated ion channels with elevated intracellular calcium—all contributing to enhanced intracellular water mobility (36, 37). Additionally, enhanced aerobic glycolysis (Warburg effect) led to lactic acid accumulation and intracellular acidosis, which could alter water–membrane interactions and modulate membrane transporter activity (38, 39), further increasing water diffusivity. Thirdly, ER+ and PR+ tumors were frequently associated with a fibrotic or desmoplastic stromal reaction that further limited water diffusion. In contrast, ER− and PR− tumors, particularly TN cancers, typically presented with prominent lymphocytic infiltration and reduced stromal fibrosis (40–42). This might contribute to the looser tissue structure, larger intercellular spaces, and increased diffusivity observed in our studies.

In this study, T_d_-dMRI–derived parameters did not differ significantly between high and low Ki-67 expression groups, which appeared inconsistent with some prior reports suggesting a correlation between diffusion metrics and proliferative activity (28). This discrepancy may stem from a mismatch between the biological role of Ki-67, a nuclear marker of proliferative activity, and the microstructural features probed by T_d_-dMRI, such as cell size, extracellular architecture, and membrane properties. Furthermore, the relatively small sample size of each Ki-67 subgroup may have limited the statistical power to detect subtle microstructural differences, even if they were present.Another important consideration was the potential influence of tumor stage on the observed associations between T_d_-dMRI parameters and molecular subtypes. Because Luminal A tumors in our study were predominantly T1 stage, while HER2-enriched and TN subtypes more frequently presented at later stages, part of the observed T_d_-dMRI differences may be influenced by variations in tumor burden or microenvironmental remodeling associated with progression. Although our exploratory analysis did not identify significant differences in key T_d_-dMRI parameters between early-stage (T1) and more advanced (≥ T2) lesions, we acknowledged that the influence of tumor stage cannot be fully excluded due to the limited sample size. Future studies with more balanced stage distributions and dedicated stage-adjusted analyses would be essential to validate subtype-specific imaging signatures.

A major highlight of this study was the findings that T_d_-dMRI showed potential for distinguishing HER2-low tumors from both HER2-zero and HER2-positive breast cancers. In particular, T_d_-dMRI-derived ADC metrics—most notably ADC_30ms_ and ADC_50ms_—demonstrated promising discriminatory ability for separating HER2-zero from HER2-low tumors, with ADC_30ms_ yielding the highest AUC (0.898) in our cohort. In our study, HER2-zero tumors demonstrated significantly higher ADC values across multiple diffuse ion time scales (ADC_50ms_, ADC_30ms_, ADC_25Hz_, ADC_50Hz_) compared to HER2-low lesions. This likely resulted from differences in tissue architecture, despite similar cellularity and cell diameter between the two groups. HER2-low tumors might possess a denser extracellular matrix rich in fibrotic components and more compact cellular arrangements, leading to restricted intercellular water diffusion (43). Additionally, HER2-low tumors—though traditionally considered HER2-negative—might retain partial HER2-driven proliferative signaling, which could alter intracellular architecture, such as cytoskeletal organization or organelle density, thereby further reducing diffusivity (44). In contrast, HER2-zero tumors exhibited looser cell packing and a less fibrotic stroma, facilitating greater water mobility and resulting in higher ADC values. Interestingly, HER2-positive tumors exhibited higher ADC (ADC_50ms_, ADC_30ms_, ADC_25Hz_, ADC_50Hz_) values than HER2-low tumors despite their known aggressiveness and larger cell diameters. This seemingly paradoxical observation might be partially explained by pathophysiological features associated with HER2 overexpression. HER2-positive tumors have been reported to show upregulated vascular endothelial growth factor (VEGF)-mediated angiogenesis, elevated microvascular density and permeability, and a higher likelihood of intratumoral necrosis or cystic degeneration, which could contribute to the diffusion characteristics observed in this study (45, 46). Elevated perfusion and interstitial fluid may increase extracellular volume, partially counteracting the restrictive effects of high cellularity and thereby elevating ADC values. Moreover, aggressive HER2-positive tumors tend to outgrew their blood supply, resulting in central necrosis and cystic degeneration. Loss of viable cells and membrane breakdown in these regions further increases water mobility, contributing to higher ADC values. Collectively, these findings suggested that T_d_-dMRI metrics can capture clinically relevant microstructural differences among HER2 expression subgroups and may assist in more refined imaging-based subtyping and therapeutic stratification, especially as HER2-low tumors become candidates for novel antibody-drug conjugates (e.g., trastuzumab deruxtecan).

This study had several limitations. First, it was conducted at a single institution with a relatively limited sample size, particularly in luminal A and HER2-zero breast cancers, and further validation in larger, multi-center cohorts should be warranted. Second, due to the resolution and signal-to-noise constraints inherent to T_d_-dMRI and considering that larger tumors better reflect underlying biological characteristics and intratumoral heterogeneity, this study included only lesions greater than 1 cm in diameter to enhance measurement accuracy. Third, although T_d_-dMRI parameters were interpreted in the context of known breast cancer microstructural features, their biological underpinnings remain inferential rather than directly validated. Future studies should correlate T_d_-dMRI metrics with more comprehensive histological analyses (e.g., quantitative assessment of cellularity, stromal fraction from whole-slide digital pathology) to establish a more robust biological basis for these imaging biomarkers. Finally, this study focused on pre-treatment imaging data. Longitudinal assessment of T_d_-DWI changes during or after therapy may offer additional value in predicting treatment response or prognosis and should be explored in future studies.

In conclusion, we demonstrated the diagnostic potential of T_d_-dMRI metrics—including ADC values derived under varying diffusion times and oscillation frequencies, as well as microstructural parameters such as D_in_, cellularity, and cell diameter— in differentiating breast cancer molecular subtypes and capturing underlying tumor biology and microstructural heterogeneity associated with hormone receptor and HER2 status. T_d_-dMRI showed promise as a noninvasive tool for subtype characterization and might offer auxiliary value in stratifying patients, guiding treatment decisions, and predicting tumor behavior in breast cancer. However, further validation in larger, multicenter, and longitudinal studies was warranted.

## Data Availability

The raw data supporting the conclusions of this article will be made available by the authors, without undue reservation.
