# Correction: Time-dependent diffusion MRI for noninvasive molecular subtype differentiation and biological correlation in breast cancer: emphasizing the emerging three-tier HER2 classification

**DOI:** 10.3389/fonc.2026.1892849

**Published:** 2026-06-09

**Authors:** Mingzhe Xu, Kuiyuan Liu, Shouning Zhang, Haotian Li, Renzhi Zhang, Chunmiao Xu, Junhui Yuan, Yue Wu, Dan Wu, Xuejun Chen, Jinrong Qu

**Affiliations:** 1 The Department of Radiology, The Affiliated Cancer Hospital of Zhengzhou University & Henan Cancer Hospital, Zhengzhou, China; 2 The Department of Biomedical Engineering, College of Biomedical Engineering & Instrument Science, Zhejiang University, Hangzhou, Zhejiang, China; 3 The Department of Radiology, National Cancer Center/National Clinical Research Center for Cancer/Cancer Hospital, Chinese Academy of Medical Sciences and Peking Union Medical College, Beijing, China

**Keywords:** breast cancer, HER2 expression, molecular subtypes, subtype differentiation, time-dependent diffusion-weighted imaging

The funder “Henan Province Central Plains Talent Program (Nurturing talent Series)], No.RS0011” was erroneously omitted. The **Funding** statement has been updated to:

“The author(s) declared that financial support was received for this work and/or its publication. This work has supported by National High Level Hospital Clinical Research Funding, National Cancer Cencer Climbing Fund (No. NCC202416005) and Henan Province Central Plains Talent Program (Nurturing talent Series)], No.RS0011.”

The original version of this article has been updated.

